# Urotropin in Anterior Poliomyelitis and Meningeal Infections Generally

**Published:** 1911-03

**Authors:** 


### Urotropin in Anterior Poliomyelitis and Meningeal Infections Generally

The advocates of the prophylactic and therapeutic use of urotropin in anterior poliomyelitis and meningeal infections generally are constantly growing in number. Clinical experience appears to bear out the logical deductions of Cushing and Crowe (*Johns Hopkins Medical Bulletin,* April, 1908, and April, 1909), who discovered the excretion of urotropin in the cerebrospinal fluid and first suggested its use in this field.

Among recent writers on the subject are: Dr. Andrew L. Skoog, Kansas City, Mo., Assistant Professor of Neurology, University of Kansas, who suggests that in anterior poliomyelitis the drug be given in as large doses as is safe over a period of a few hours and then discontinued for about twenty-four hours. His method is to give from 0.12 to 0.24 gramme to a child two to four years of age, giving a dose every hour until three doses have been given. No more is then given until the following day at the same hour, when it is repeated in the same manner. This repetition may be employed as long as acute symptoms persist. To adults he gives from 3 to 8 grammes of the drug during the daily three to four-hour period of its administration.—(*Journal of the A.M.A.,* November 19, 1910.)

Dr. C. F. Clowe, Schenectady, N. Y., writing on the "Diagnosis and Treatment of Anterior Poliomyelitis" (*N. Y. State Journal of Medicine,* November, 1910), describes eighteen cases which have been reported to the health department of his city last summer. Three of the most severe were treated with urotropin and in these the recovery was more complete than was anticipated. Dr. Alfred Friedlander, Cincinnati, O., in an article on

"Acute Anterior Poliomyelitis" (*Interstate Medical Journal.* November, 1910), reviews the most important contributions to the literature in 1910 and concludes that the early and free use of urotropin is certainly advisable. Editorial in *Colorado Medicine,* October, 1910, dealing with epidemic poliomyelitis states that in addition to the usual symptomatic treatment it is strongly recommended by several clinicians that urotropin be given as early as possible and in generous doses during the acute stages. Editorial in the *Denver Medical Times and Utah Medical Journal* November, 1910, under the heading of "New Facts about Poliomyelitis" emphasizes the fact that urotropin tends to sterilise the cerebrospinal fluid, and that if given early in full doses may prove to be of great value.

Dr. J. Ibrahim, Chief Physician of the Gisela Children's Hospital in Munich, Germany, reports in *Medizinische Klinik,* 1910, No. 48, the results of extensive experiments with urotropin in the treatment of serous and suppurative meningitis in children and advocates on the strength of his observations that urotropin be used invariably in all forms of the diseases mentioned. Dr. Walter Brem, Chief of the Medical Clinic, Ancon Hospital, Ancon, Canal Zone, discusses in the *N. Y. Medical Journal* of October 22, 1910, the use of urotropin in meningeal inflammation and reports a case of meningococcus meningitis in which he used it. The exertion of urotropin in the cerebrospinal fluid having been experimentally established by Cushing and Crowe, the author believes that this drug should be used promptly in all cases in which meningitis is a possible complication. When meningitis is already present it is indicated as an adjunct to the treatment or as a substitute for Flexner's serum when the latter cannot be obtained.

